# Clinical Applicability of Visible Light‐Mediated Cross‐linking for Structural Soft Tissue Reconstruction

**DOI:** 10.1002/advs.202300538

**Published:** 2023-07-09

**Authors:** Gretel Major, Alessia Longoni, Jeremy Simcock, Nicholas J Magon, Jessica Harte, Boushra Bathish, Roslyn Kemp, Tim Woodfield, Khoon S Lim

**Affiliations:** ^1^ Department of Orthopaedic Surgery and Musculoskeletal Medicine Centre for Bioengineering & Nanomedicine University of Otago Christchurch 8011 New Zealand; ^2^ Department of Surgery University of Otago Christchurch 8011 New Zealand; ^3^ Centre for Free Radical Research Department of Pathology and Biomedical Science University of Otago Christchurch 8011 New Zealand; ^4^ Jacqui Wood Cancer Centre Division of Cellular Medicine Ninewells Hospital and Medical School University of Dundee Dundee Scotland DD2 1GZ UK; ^5^ Department of Microbiology and Immunology University of Otago Dunedin 9016 New Zealand; ^6^ Light‐Activated Biomaterials Group School of Medical Sciences University of Sydney Sydney 2006 Australia

**Keywords:** fat grafting, patient‐derived tissues, photocross‐linking, structural control

## Abstract

Visible light‐mediated cross‐linking has utility for enhancing the structural capacity and shape fidelity of laboratory‐based polymers. With increased light penetration and cross‐linking speed, there is opportunity to extend future applications into clinical spheres. This study evaluated the utility of a ruthenium/sodium persulfate photocross‐linking system for increasing structural control in heterogeneous living tissues as an example, focusing on unmodified patient‐derived lipoaspirate for soft tissue reconstruction. Freshly‐isolated tissue is photocross‐linked, then the molar abundance of dityrosine bonds is measured using liquid chromatography tandem mass spectrometry and the resulting structural integrity assessed. The cell function and tissue survival of photocross‐linked grafts is evaluated ex vivo and in vivo, with tissue integration and vascularization assessed using histology and microcomputed tomography. The photocross‐linking strategy is tailorable, allowing progressive increases in the structural fidelity of lipoaspirate, as measured by a stepwise reduction in fiber diameter, increased graft porosity and reduced variation in graft resorption. There is an increase in dityrosine bond formation with increasing photoinitiator concentration, and tissue homeostasis is achieved ex vivo, with vascular cell infiltration and vessel formation in vivo. These data demonstrate the capability and applicability of photocrosslinking strategies for improving structural control in clinically‐relevant settings, potentially achieving more desirable patient outcomes using minimal manipulation in surgical procedures.

## Graphical abstract

This work introduces visible light‐mediated cross‐linking of heterogenous living tissues and demonstrates the capability and applicability of these strategies for improving structural control in clinically‐relevant settings. Patient‐derived lipoaspirate is photocross‐linked and dityrosine bond formation, structural integrity, vascularization, and tissue viability are assessed in vitro, ex vivo and in vivo. These data provide a conclusive example of the opportunities of photocross‐linking technologies for delivering structural support to heterogenous patient‐derived materials to ultimately achieve more desirable patient outcomes.

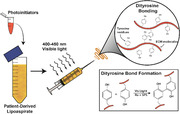

## 1 Introduction

Light‐mediated cross‐linking technologies are becoming extensively used in bioengineering and biology fields, showing breadth in utility for the fabrication of hydrogels, drug and growth factor delivery systems and to obtain spatial and temporal control in three‐dimensional (3D) biofabrication approaches.^[^ ^1^, ^2^, ^3^, ^4^, ^5^, ^6^ ^]^ Current applications predominantly focus on the use of homogeneous, laboratory‐based materials, such as purified native proteins,^[^ ^7^, ^8^, ^9^, ^10^, ^11^ ^]^ chemically‐functionalized macromers^[^ ^12^, ^13^ ^]^ and other synthetic polymers.^[^ ^5^, ^14^ ^]^ However, given the increased photocross‐linking speed, cytocompatibility and penetration depth of visible light‐based systems^[^ ^12^, ^15^ ^]^ there is opportunity to broaden the use of these technologies to other more complex materials and problems with greater clinical relevance, such as patient tissues containing cells and heterogeneous extracellular matrix (ECM). An innovative approach for which this could be applicable is improving tissue retention after fat grafting procedures, through modifying the structural properties of patient‐derived lipoaspirate.

Lipoaspirate is cannula‐harvested adipose tissue used as a filling material in autologous fat grafting procedures to repair soft tissue defects in a broad range of clinical indications, such as burn and traumatic scars, congenital defects, cushioning implants and joints, and following ablative surgical procedures (e.g., tumor resection).^[^ ^16^, ^17^, ^18^, ^19^ ^]^ For many of these indications, soft tissue reconstruction is necessary to prevent negative effects on cosmesis and the functional deficits caused by scar contracture such as poor dermal support and reduced range of movement.^[^ ^20^, ^21^ ^]^ Nevertheless, fat grafting procedures suffer from unpredictable long‐term clinical outcomes including variable resorption rates and potential side effects (e.g., tissue necrosis, oil cysts, calcifications), which are exacerbated in larger volume grafts (e.g., breast reconstruction after mastectomy).^[^ ^17^, ^22^, ^23^ ^]^ The inability to predict the outcomes of fat grafting patients often translates into a need for multiple surgeries to replace the resorbed or necrotic tissue. The main reasons for poor graft survival and high resorption rates is the limited structural fidelity of lipoaspirate as a filling material, which when injected into the defect site, does not retain its shape and frequently coalesces into large unstructured globules.^[^ ^24^, ^25^ ^]^ In particular, large tissue deposits have a low surface area‐to‐volume ratio and therefore a low graft‐to‐recipient interface, resulting in limited nutrient diffusion during tissue revascularization, leading to the formation of necrotic tissue (which is eventually reabsorbed by the body).^[^ ^24^, ^25^ ^]^ Improving the structural capabilities of lipoaspirate would allow for greater shape retention of the thin micro‐ribbon fibers that are injected into the defect space,^[^ ^17^ ^]^ preserving the graft‐to‐recipient interface, and resulting in a reduced distance for nutrient diffusion and vascular in‐growth.

A light‐mediated cross‐linking method for this application could be used to overcome some of the drawbacks evident in other approaches to improving fat grafting outcomes. The predominant and most relevant strategies that have previously been used to control the shape and structure of tissue grafts, have been through encapsulating and differentiating mesenchymal stromal cells (e.g., MSCs, or ADSCs) in hydrogel matrices, or forming lipoaspirate composites through addition of supportive polymer matrices.^[^ ^17^, ^26^, ^27^, ^28^, ^29^, ^30^ ^]^ The employment of hydrogel support matrices in both instances requires significant manipulation, decellularization or functionalization of tissue components but allows for improved graft structure and offers the potential for further spatial control through application of biofabrication approaches. Nevertheless, the main limitations of these strategies are their restricted scalability, immature adipose tissue products (due to reliance on cellular differentiation), the use of non‐clinically approved materials and the need for an invasive surgical implantation (rather than the traditional percutaneous approach as currently used in fat grafting).^[^ ^17^ ^]^ Unlike these other strategies, applying a light‐based crosslinking approach to patient‐derived lipoaspirate before it is re‐implanted as a filling material would involve minimal modification of the surgical procedure and provide a more direct route for clinical translation.

Advances in hydrogel systems, especially those involving decellularized extracellular matrix (dECM) materials, provide a promising basis for the application of the proposed light‐mediated cross‐linking system to lipoaspirate by initiating covalent bond formation between ECM molecules present within the living patient‐derived tissue.^[^ ^7^, ^8^, ^9^, ^31^, ^32^ ^]^ The proposed system works simply through addition of photoinitiatiors, tris(bipyridine)ruthenium(II) chloride (Ru) and sodium persulfate (SPS), to polymers containing phenolic residues (e.g., tyrosine) and then irradiation with visible light at 400 – 500 nm.^[^ ^4^, ^33^ ^]^ Covalent bond formation occurs through photolysis of Ru^2+^ into Ru^3+^ (high oxidation state), which then oxidizes phenolic residues (e.g., tyrosine) on polymer chains.^[^ ^33^ ^]^ These oxidized phenolic residues are converted into tyrosyl free radicals, which then form biphenol bonds (e.g., dityrosine) with nearby tyrosine groups. As demonstrated for hydrogel applications, the clear advantages of this photocross‐linking technique include high cross‐linking speed and penetration depth, wide tailorability in resulting material properties and cytocompatibility at low photoinitiator concentrations with a range of cell types.^[^ ^5^, ^9^, ^12^, ^15^, ^31^ ^]^ Due to the presence of tyrosines within ECM molecules, this strategy has already been used to generate dECM hydrogels, which demonstrate high shape‐fidelity and material control when applied to biofabrication platforms.^[^ ^31^ ^]^ Given that lipoaspirate also contains a dense ECM network with abundant tyrosine residues throughout,^[^ ^34^ ^]^ this photocross‐linking system may also facilitate dityrosine bond formation between lipoaspirate ECM molecules and thereby improve the structure of the tissue.

The primary objective of this study is to investigate the clinical applicability of visible light‐mediated cross‐linking (photocross‐linking) for obtaining structural control in unmodified living patient tissues. As there is a clear need to develop strategies to improve the utility of lipoaspirate for fat grafting applications (due to the weak mechanical properties of lipoaspirate resulting in grafts with poor shape maintenance, low surface area‐to‐volume ratio and therefore limited vascularization), detailed focus is given to improving the structural fidelity of lipoaspirate for enhanced tissue integration and structural control in fat grafting procedures. Our specific aims were to: 1) identify and quantify the bonding mechanism in photocross‐linked patient tissues, 2) investigate changes in the base properties of photocross‐linked tissues, 3) measure the response of patient‐derived tissues to photocross‐linking, 4) evaluate the success of photocross‐linked graft transplantation in vivo, 5) measure the extent of vascularization within transplanted grafts, and 6) evaluate graft shape integrity under controlled delivery.

We show that by applying the proposed photocross‐linking strategy to clinical fat grafting procedures between lipoaspirate harvest and implantation of the graft, there is good potential to have greater control over the shape integrity and stability of the grafts once implanted.

## 2 Results and Discussion

In this study, a visible light photocross‐linking strategy was optimized for patient‐derived lipoaspirate to provide greater controlled delivery, tissue retention and augmented cell survival when implanted. Our approach involved collection of fresh patient‐derived lipoaspirate from fat grafting procedures and photocross‐linking the patient material in syringes or moulds; followed by covalent bond evaluation, material characterization, ex vivo culture, inflammatory cytokine analysis, in vivo transplantation, or structural analyses – each of which is discussed separately below.

### 2.1 Bonding Mechanism & Quantification of Photocross‐linked Tissue

**Figure 1:**
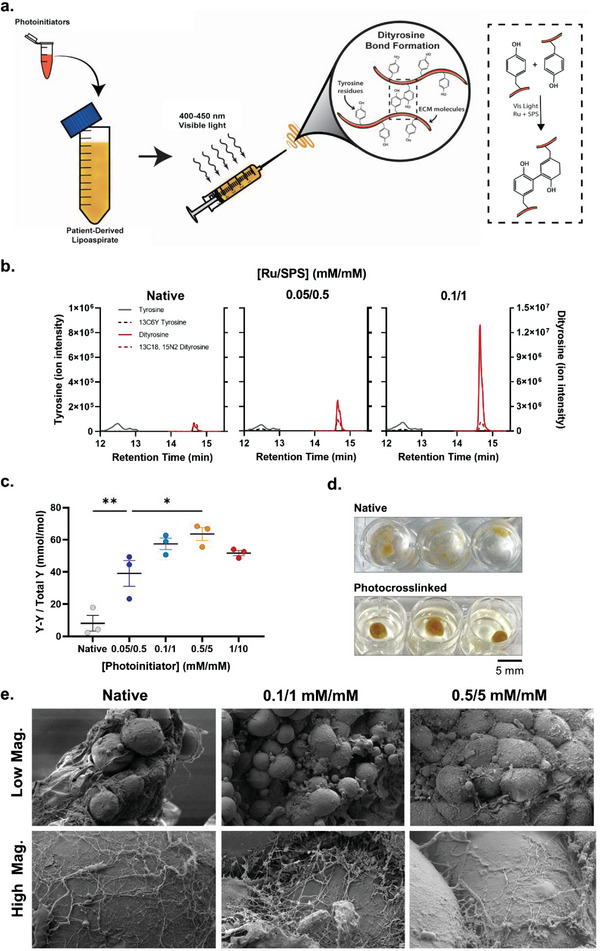
Covalent bond formation, quantification, and visualization in photocross‐linked lipoaspirate. a) Schematic overview of photocross‐linking approach and mechanism of biphenol bond (e.g., dityrosine) formation at a molecular level. b) Typical LC‐MS/MS chromatograms obtained for the analysis of tyrosine (grey lines) and dityrosine (red lines) in ECM isolated from photocross‐linked samples. Chromatograms for isotopically‐labelled analytes (dotted lines) are superimposed on unlabeled counterparts (solid lines). c) Quantification of dityrosine (Y‐Y) abundance in isolated lipoaspirate ECM samples calculated using standard calibration curves normalized to isotopically‐labelled internal standards. Values presented are the means of three individual patients (with each patient sample performed in triplicate). Error bars represent ± standard error. Asterisks denote significant differences as detected by one‐way ANOVA, *n* = 9, * *p*< 0.05, ** *p*<0.01. d) Macro images of native and photocross‐linked lipoaspirate samples immersed in PBS. e) SEM images of native and photocrosslinked lipoaspirate samples at low (≈300 ×) and high (≈3000 ×) magnification, qualitatively showing maintained adipocyte morphology and fibril branching in cross‐linked samples.

To generate biphenol bonds (e.g., dityrosine) between ECM proteins present within the patient‐derived tissue, lipoaspirate was mixed with photoinitiators (Ru and SPS) and irradiated with visible light (**Figure** **1**a). The molar abundance of tyrosine and dityrosine ions was quantified and reported by liquid chromatography tandem mass spectrometry (LC‐MS/MS) with retention times of ≈12.5 and 14.7 min, respectively (Figure 1b). Previous studies have demonstrated the presence of covalent phenolic bonds from photocross‐linking by SDS‐PAGE and Western blotting,^[^ ^7^, ^33^, ^35^ ^]^ fluorescence detection,^[^ ^7^, ^36^, ^37^, ^38^, ^39^ ^]^ and finally comparing analyte retention times to standards using LC‐MS/MS.^[^ ^7^, ^11^ ^]^ However, this study provides increased value by using a stable isotope dilution LC‐MS/MS method to quantify the molar abundance of dityrosine bonds formed by photocross‐linking. In native lipoaspirate (containing no photoinitiators), the ratio of dityrosine to total tyrosine detected was ≈8.1 mmol mol^−1^, which increased linearly (R^2^ = 0.978) with photoinitiator concentration to 57.5 mmol mol^−1^ at 0.1/1 mm mm ^−1^ (Ru/SPS) (Figure 1c). Above this photoinitiator concentration, dityrosine bond formation began to plateau, decreasing to 51.7 mmol mol^−1^ at 1/10 mm mm.^−1^ This reduced bond formation at higher photoinitiator concentrations is likely due to an overabundance of energy input into the system during the initiation phase, resulting in termination of cross‐linking without complete propagation (i.e., generation of cross‐links).^[^ ^4^, ^40^ ^]^ To confirm this trend, samples were photocross‐linked with higher photoinitiator concentrations (2/20 mm mm ^−1^), demonstrating a further reduction in dityrosine ions, to 24.8 mmol mol^−1^. Further semi‐quantitative analysis of trityrosine abundance (relative to the heavy dityrosine standard) revealed similar trends to dityrosine species, suggesting that the reduced level of dityrosine bonds at higher photoinitiator concentration was not due to further reactivity of dityrosine species into more complex tyrosine products, as detected in resilin (Figure S2, Supporting Information).^[^ ^41^, ^42^ ^]^ Further studies looking at the quantification of other phenolic bonds (e.g., tryptophan and phenylalanine) and tyrosine oligomers may help in further understanding covalent bond formation due to photocross‐linking in these complex tissues;^[^ ^43^ ^]^ however, this is currently limited by the need to develop representative standards of analytes of interest for quantification via LC‐MS/MS.

When viewing the structural changes associated with bond formation on a macro level, photocrosslinked lipoaspirate appeared to be stitched together into structurally confined forms, which retained shape integrity when immersed in PBS (Figure 1d). From qualitative observations at a finer micro scale, clear ECM fibrils could be identified among adipocyte cells in both native and photocross‐linked samples (Figure 1e). The ECM fibrils appeared more branched and tightly bound to the cells when the lipoaspirate was photocross‐linked using this strategy (Figure 1e). These structural changes are consistent with previous work in peptide hydrogels, where fibrils became more branched and varied in diameter when photocross‐linked, and together provide evidence for the capability of photocross‐linking for bonding native ECM molecules together and forming a more compact tissue.^[^ ^38^ ^]^

### 2.2 Material Characterization of Photocross‐linked Tissue

**Figure 2:**
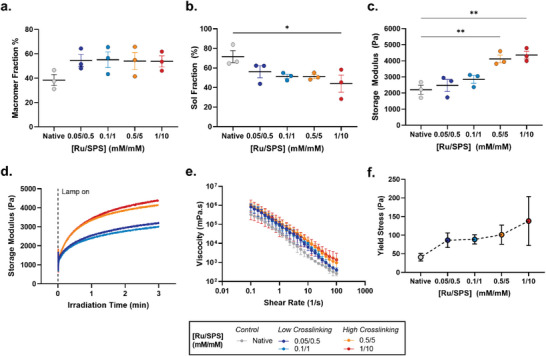
Physiochemical and rheological characterization of photocross‐linked lipoaspirate. a) Percentage macromer fraction, and b) sol fraction of photocross‐linked lipoaspirate as observed via mass‐loss analysis. Oscillatory rheological assessment of complex shear modulus c) after 3 min and d) observed over 3 min of visible light irradiation. e) Lipoaspirate viscosity profile with relation to increased shear rate, and f) yield stress behavior. Values presented are the means of three individual patients (with each patient sample performed in triplicate). Error bars represent ± standard error. Asterisks denote significant differences as detected by one‐way ANOVA, *n* = 9, * *p*< 0.05, ** *p*<0.01.

A thorough rheological and tissue retention investigation was conducted to understand the effects of photocross‐linking on the base tissue properties of patient‐derived lipoaspirate. For mass‐loss studies, samples were treated with isopropanol to largely remove the lipid portion of the samples, and to therefore achieve more accurate determination of macromer fraction and sol fraction. As expected, the macromer fraction was equal for all photocross‐linked groups at ≈55%, suggesting that lipids made up approximately half of the initial tissue weight and validates equal starting material in all samples (**Figure** **2**a). The uncross‐linked control had a reduced macromer fraction at 40% due to tissue dispersion and loss (≈15%) during the isopropanol treatment (Figure 1d and Figure 2a). Similar findings were also observed in the sol fraction where there was a general trend toward a lower sol fraction with increased photocross‐linking (Figure 2b). These findings are consistent with other studies focused on photopolymerization in hydrogels,^[^ ^12^, ^15^ ^]^ however in this application the concentration of photoinitiators required was 10‐fold lower than previously reported. Taken together, these data demonstrate that photocross‐linking lipoaspirate with low concentrations of photoinitiators, helps confine and compact the tissue into a robust shape for better structural retention, and prevention of tissue dispersion and subsequent loss.

The compromised structure of native lipoaspirate observed undeniably coincides with reduced tissue mechanical properties.^[^ ^44^ ^]^ Native lipoaspirate had a storage modulus of ≈2000 Pa at room temperature, which could be increased up to 2‐fold with photocross‐linking at ≈4000 Pa (Figure 2c). The increased stiffness of photocross‐linked grafts demonstrates a restoration of mechanical properties toward native excised adipose tissue, which is up to 10‐fold stiffer than native lipoaspirate.^[^ ^44^, ^45^, ^46^ ^]^ Furthermore, there was a clear difference in photocross‐linking behavior and final storage modulus obtained between the low (blue and teal data points) and high (orange and red data points) photocross‐linking groups (Figure 2c), indicating the capacity of photocross‐linking for controlling lipoaspirate tissue properties by manipulating different levels of ECM cross‐linking. While there is a trend toward an increased storage modulus with low photocross‐linking, it appears that tuning the local microenvironment structure (through dityrosine cross‐linking of the ECM) contributes more greatly to the structural integrity of the graft, compared with an enhancement of bulk mechanical properties (Figure 1b,c and Figure 2c). This is further supported by the reduction of sol fraction measured at low photocross‐linking intensities (Figure 2b). For all groups, the majority of photocross‐linking occurred within the first minute of visible light exposure, as demonstrated by the early spike in storage modulus and reduction in fiber diameter, which then plateaued with negligible changes over 3 min (Figure 2d; Figure S9, Supporting Information).This photocross‐linking pattern is consistent with previous studies using this photoinitiator system in gelatin hydrogels, demonstrating a stable sol fraction after 60 sec of light exposure.^[^ ^12^ ^]^ The viscosity of the patient material also increased with photoinitiator concentration, resulting in a yield stress 2‐fold and up to 3‐fold higher than native lipoaspirate for low and high cross‐linking conditions respectively (Figure 2e,f). This rheological characterization was expected when considering the trend in dityrosine bond formation (Figure 1c), and reaffirms that bond formation, as a product of photocross‐linking, results in more stable lipoaspirate tissue mechanics. For clinical delivery applications, an understanding of how these increased mechanical properties may affect cell survival is an important consideration as previous rheological evaluation of lipoaspirate extruded from cannulas of variable diameters has demonstrated clear differences in lipoaspirate retention in vivo.^[^ ^45^ ^]^

### 2.3 Ex Vivo Response of Photocross‐linked Patient‐Derived Tissues

**Figure 3:**
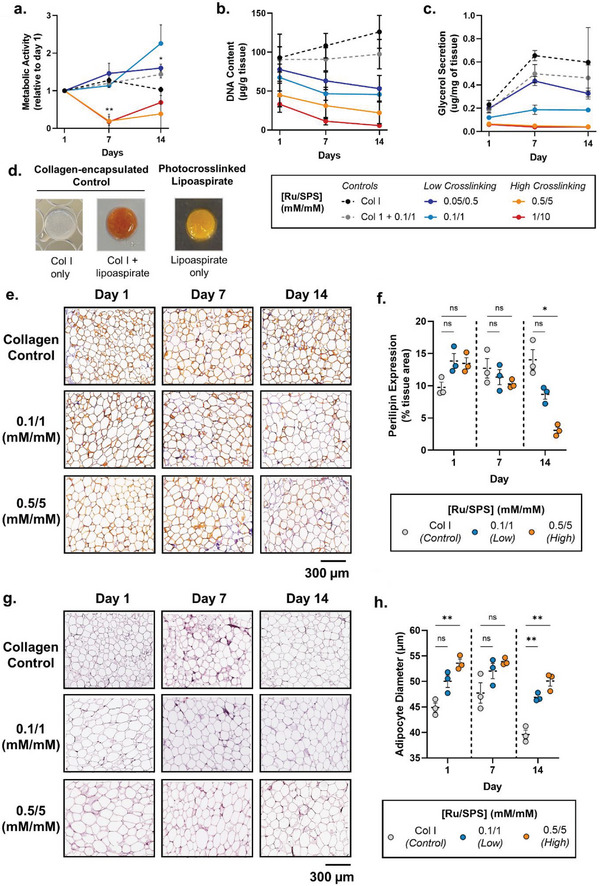
Functional and histological characterization of the lipoaspirate tissue response to photocross‐linking. a) Metabolic activity (normalized to DNA‐content), b) DNA‐content and c) glycerol secretion (normalized to tissue weight) of photocross‐linked lipoaspirate cultures over 14 days. d) Macro‐images of collagen‐encapsulated lipoaspirate controls and photocross‐linked lipoaspirate. e) Representative perilipin immunohistochemistry sections at 10× magnification, and f) perilipin expression quantification of low (0.1/1 mm mm ^−1^ Ru/SPS) and high (0.5/5 mm mm ^−1^ Ru/SPS) photocross‐linked samples over 14 days. g) Representative hematoxylin and eosin‐stained sections at 10× magnification, and h) adipocyte diameter quantification of low (0.1/1 mm mm ^−1^ Ru/SPS) and high (0.5/5 mm mm ^−1^ Ru/SPS) photocross‐linked samples over 14 days. Values presented are the means of three individual patients (with each patient sample performed in triplicate). Error bars represent ± standard error. Asterisks denote significant differences as detected by two‐way ANOVA, *n* = 9,* *p*< 0.05, ** *p*<0.01.

While the cytocompatibility of visible light cross‐linking approaches has previously been investigated for cell‐encapsulated hydrogels,^[^ ^6^, ^9^, ^12^, ^47^ ^]^ the same has not been investigated for cell‐containing ex vivo tissues (such as lipoaspirate). To address this, patient‐derived lipoaspirate was photocross‐linked and cultured ex vivo over two weeks – with comparisons made to previously established collagen‐encapsulated lipoaspirate (± photoinitiators) as non‐photocross‐linked controls (**Figure** **3**d).^[^ ^48^ ^]^

There was a distinct clustering of cellular responses to photocross‐linking based on the degree of photocross‐linking (Figure 3a–c). Cells in the low photocross‐linking conditions had a similar metabolic activity profile to the collagen controls, while the high photocross‐linking conditions demonstrated a significant decrease in metabolic activity after 7 days, which gradually recovered by 14 days (P = 0.0087; Figure 3a). Interestingly, cross‐linking conditions of 0.05/0.5 mm mm ^−1^ (Ru/SPS) resulted in a significantly higher metabolic activity at 14 days compared with the collagen control (P = 0.0457), with similar trends observed in lipoaspirate and collagen‐encapsulated lipoaspirate photocross‐linked with 0.1/1 mm mm ^−1^ (Ru/SPS) (Figure 3a). This stimulation may be caused by exposure to low‐intensity visible light, which has been shown to stimulate cell metabolism in a range of cell types.^[^ ^49^, ^50^ ^]^ Nevertheless, the relatively stable metabolic activity observed across culture in most groups was expected as adipose tissue largely contains terminally‐differentiated adipocytes, which undergo minimal proliferation in this state.^[^ ^51^, ^52^ ^]^ Despite this, the DNA‐content of the collagen‐encapsulated controls increased over culture; while the low photocross‐linking intensities slowly reduced with large decreases observed at high cross‐linking intensities (Figure 3b). Interestingly, perilipin expression was not significantly different between the controls and photocross‐linked groups at early time points, suggesting a similar abundance of live adipocytes in the samples (Figure 3e,f). The reduction in DNA content observed may therefore be partially explained by a loss of stromal cells from the photocross‐linked samples, which become attached to the bottom of the plate (not observed in the collagen controls). The collagen hydrogels encapsulated the lipoaspirate and provided a support matrix to prevent cell loss while in culture, however the photocross‐linked conditions did not have a physical barrier and therefore loosely bound cells may have detached from the sample over time. Finally, as consistent with the DNA analysis, there was a significant decline in perilipin expression by day 14 in the high photocross‐linked samples, suggesting poor adipocyte viability (Figure 3e,f). Despite the higher concentration of photoinitiators regularly used in cell‐encapsulated hydrogel applications,^[^ ^47^ ^]^ these data suggest that tissues (like lipoaspirate) only remain viable and metabolically active at low photoinitiator concentrations (where free radical production = dityrosine bond formation), while at high photoinitiator concentration (where excessive free radical production = cross‐linking termination) surplus free radicals may cause lipid peroxidation and poor tissue survival.^[^ ^12^, ^53^ ^]^

Lipid metabolism is a homeostatic function of adipose tissue involving storing triglycerides within lipid vacuoles present in adipocytes (lipogenesis) and breaking triglycerides down into glycerol and free fatty acids when needed (lipolysis).^[^ ^28^, ^52^ ^]^ To provide further detail about cellular health with photocross‐linking the lipolytic response of photocross‐linked lipoaspirate cultures was compared (Figure 3c). As in previous studies with mature adipocytes,^[^ ^48^ ^]^ there was an increase in glycerol secretion at day 7 for the controls, which then remained relatively constant until day 14 (Figure 3c). Similar trends, albeit at a smaller scale, were seen for the low cross‐linking conditions, while minimal glycerol was secreted at any time point in the high cross‐linking conditions (Figure 3c). This was reflected in the larger diameter of adipocytes with low cross‐linking and significantly larger diameter of adipocytes with high cross‐linking compared to controls (Figure 3g,h; Figure S3, Supporting Information), suggesting the accumulation of lipids within vacuoles rather than release into the media.^[^ ^28^ ^]^ The final glycerol concentration for the low crosslinking conditions (≈0.2 µg mg^−1^ tissue) and collagen‐encapsulated lipoaspirate controls (≈0.6 µg mg^−1^ tissue) was within a comparable range to published ex vivo adipose tissue models,^[^ ^28^, ^54^, ^55^ ^]^ suggesting that photocrosslinking at low intensities does not adversely affect adipose tissue homeostasis ex vivo.

### 2.4 Predicting the Immune Response to Photocross‐linked Grafts

**Figure 4:**
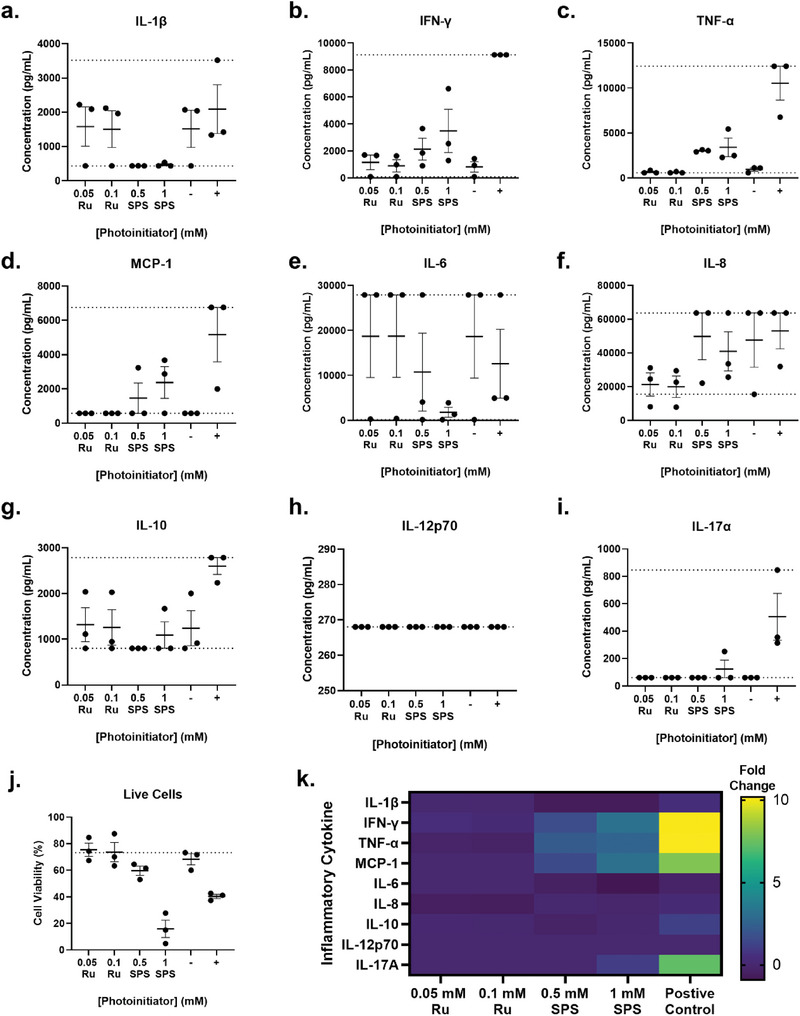
Response of peripheral blood mononuclear cell (healthy PBMC) donors to photoinitiators (ruthenium; Ru, and sodium persulfate; SPS). LegendPlex assay to detect production of inflammatory cytokines including a) IL‐1*β*, b) IFN‐*γ*, c) TNF‐*α*, d) MCP‐1, e) Il‐6, f) IL‐8, g) IL‐10, h) IL‐12p70 and i) IL‐17*α*. j) Cell viability as measured using propidium iodide gating. Values presented are the means of three individual donors, with no significant differences detected between groups as detected by the Kruskall‐Wallis one‐way ANOVA. Error bars represent ± standard error. k) Heat map of fold‐change in cytokine production compared with the negative control.

To anticipate any clinical immune response to photocross‐linking and to provide a more detailed preclinical evaluation of photocross‐linked grafts, cytokine bead arrays were used to assess the production of inflammatory cytokines by peripheral blood mononuclear cells (PBMCs) in response to the photoinitiations used for photocross‐linking. There was a large variation in inflammatory cytokine production across the three healthy PBMC donors screened, with no significant differences detected based on exposure to the photoinitiators at the different concentrations used (**Figure** **4**). Exposure to Ru at 0.05 and 0.1 mm did not modulate secretion of any of the cytokines, mimicking the same levels as the negative control (Figure 4). Furthermore, Ru did not affect PBMC cell viability, with over 73% of cells viable – ≈5% more than the negative control (Figure 4j). This suggests that Ru present within the lipoaspirate grafts will not cause an immune response or reduce cell viability, which is a particularly important finding given that Ru is recycled during bond formation (moving between a baseline (Ru^2+^) and excited state (Ru^3+^)), and therefore may take time to clear from the tissue.^[^ ^4^, ^33^ ^]^

Unlike Ru, SPS is the rate limiting factor during bond formation and is depleted in the reaction,^[^ ^7^, ^33^ ^]^ suggesting that less SPS should be present within the photocross‐linked lipoaspirate grafts. Investigation of the photocross‐linking reaction kinetics revealed a 15‐fold decrease in SPS concentration after photocross‐linking and the resulting leachates from native fibrinogen hydrogels appeared non‐toxic to cells.^[^ ^7^ ^]^ SPS treatment at 0.5 and 5 mm resulted in a reduction in IL‐1*β*, IL‐6 and IL‐10 levels, and an increase in IFN‐*γ*, TNF‐*α* and MCP‐1 compared to the negative control (Figure 4). There were minimal changes to IL‐8, IL‐12p70 and IL‐17 (Figure 4). None of these changes were significantly different, with all changes lower than the anti‐CD3/CD28 stimulated positive controls (Figure 4). Furthermore, the magnitude of these changes in inflammatory cytokine production may also be complicated by the poor viability of PBMCs when treated with 1 mm SPS; with only 15.89% viable (4.3‐fold less than the negative control) (Figure 4j).

Despite acknowledgement of the reduced concentration of SPS likely in the grafts, screening of SPS and Ru alone in this study was an important step to understand how each component contributes individually to the immune response, as this has not been done previously. Furthermore, while the positive control in this experiment was stimulated T cells, monocytes present within the PBMC cultures were important for mimicking the native fat environment, given that macrophages are the main immune cells present within autologous fat grafts.^[^ ^56^ ^]^ This study therefore provides an appropriate surrogate for understanding the types of cytokine changes that may occur with stimulated T‐cells and macrophages with addition of photoinitiators to grafts. Further studies of reaction products may provide utility for understanding the biosafety profile of these grafts for clinical translation.

### 2.5 Graft Transplantation & Tissue Evaluation In Vivo

**Figure 5:**
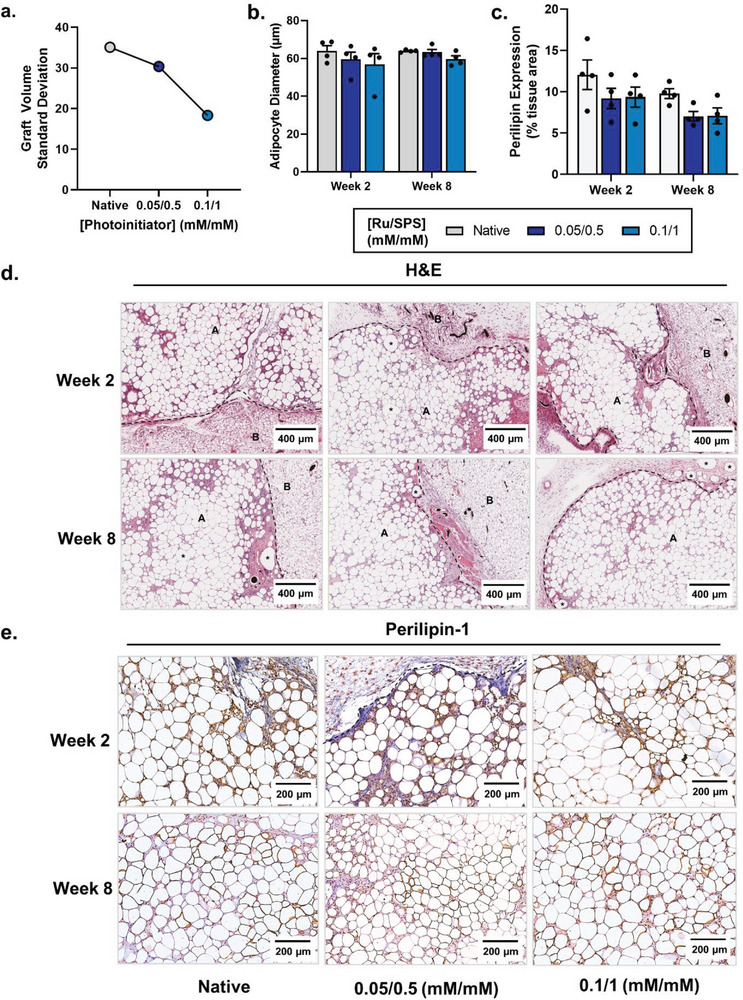
Transplantation and tissue evaluation of photocrosslinked grafts in vivo. a) Graft volume standard deviation after eight‐weeks (*n* = 4). b) Mean adipocyte diameter and c) perilipin expression quantification of photocrosslinked grafts after two and eight weeks. Values presented are the means of four mice implanted with the same patient sample (with quantification performed on triplicate slides per graft). Error bars represent ± standard error. Representative d) hematoxylin and eosin‐stained sections and e) perilipin immunohistochemistry sections, at 10× magnification after two and eight weeks. A: lipoaspirate graft of human origin; B: surrounding host subcutaneous adipose tissue; dotted line: graft margin; *: oil cysts.

To better understand the effects of photocross‐linking lipoaspirate on fat grafting clinical outcomes, patient‐derived lipoaspirate was photocross‐linked and implanted subcutaneously in mice. As expected based on previous studies,^[^ ^56^, ^57^, ^58^ ^]^ over the eight‐week implantation both the photocross‐linked and native lipoaspirate grafts partially resorbed, with ≈78% of the implanted volume remaining at the end of the study regardless of experimental group (Table S4, Supporting Information). Photocross‐linking resulted in a reduction in the implant volume standard deviation within each experimental group with increasing photoinitiator concentration (**Figure** **5**a; Figure S4 and Table S4, Supporting Information); suggesting that the photocross‐linking strategy contributed to providing more predictable resorption rates, potentially by more consistently stabilizing the shape of the graft when implanted compared with native grafts.

The photocross‐linking strategy did not adversely affect the survival or integration of the grafts within the host (Figure 5). There was no significant difference in average adipocyte size between implant groups or between the two‐ and eight‐week timepoints, with ≈40% of adipocytes less than 50 µm in diameter (likely undergoing adipogenesis or remodeling^[^ ^59^ ^]^) and the mean adipocyte diameter measured at ≈60 µm (Figure 5b; Figure S5, Supporting Information). There was no difference in perilipin expression across conditions and timepoints, where viable perilipin‐positive adipocytes could be detected (as previously described^[^ ^57^, ^60^, ^61^, ^62^, ^63^ ^]^); highlighting equal survival of adipocytes between groups (Figure 5c,e). When looking more generally at tissue structure, there was no evidence of a fibrotic capsule developing in any of the groups and similar tissue structure was observed in both photocross‐linked and native controls (Figure 5d). As expected, some oil cysts could be identified within the grafts, although these were not extensive (Figure 5d). There was clear evidence of cell infiltration into all the grafts, however this tended to be more prominent at the two‐week timepoint and in the native tissue groups (Figure 5d). Interestingly, this increased cell infiltration at the two‐week time point coincided with higher perilipin within the extracellular space, suggestive of increased tissue remodeling early on following implantation (Figure 5c–e). Taken together, these data demonstrate that photocross‐linking at low photoinitiator concentrations does not impair tissue survival or integration within the host, where live adipocytes are present throughout the grafts. Longer term studies using autologous grafts would be beneficial to further understand the clinical potential of photocross‐linking for providing more predictable resorption rates.

### 2.6 Vascularization of Photocross‐linked Tissue Grafts

**Figure 6:**
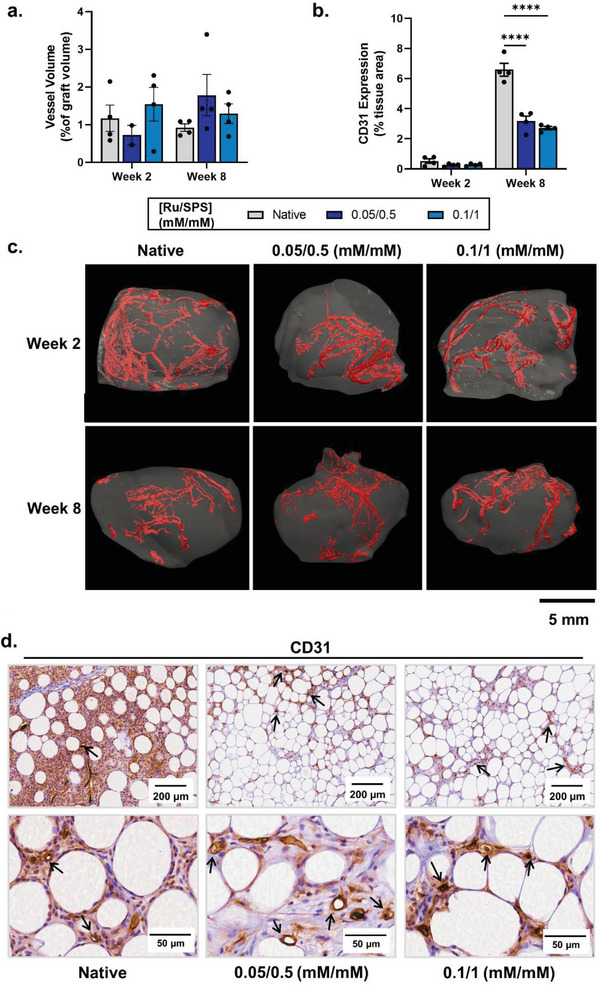
Vascularization of photocrosslinked grafts in vivo. a) Vessel volume as a percentage of total graft volume, as measured by micro‐CT (*n* = 4). b) CD31 expression quantification of photocrosslinked grafts after two and eight weeks. Values presented are the means of four mice implanted with the same patient sample (with quantification performed on triplicate slides per graft). Error bars represent ± standard error. Asterisks denote significant differences as detected by two‐way ANOVA, ****p<0.0001. c) Representative 3D reconstructions, lateral view, and cross section (CS), of perfused vessels throughout native and photocrosslinked grafts after two and eight weeks. d) Representative CD31 immunohistochemistry sections at 10× magnification after 8 weeks. Arrows indicate vessels containing visible lumen structures.

As tissue revascularization is pivotal to graft survival and reduced resorption in the clinic, an investigation into the abundance and type of vessels formed in implanted photocross‐linked grafts was undertaken. The photocross‐linked grafts and native controls supported the formation of large functional vessels throughout the grafts, with no significant differences detected between groups or time points (**Figure** **6**a,c). This is the first study to quantify neovascularization in lipoaspirate implants in vivo using angiography, and demonstrates the accuracy of this tool to quantify vessel volumes throughout soft tissue implants, as done previously for bone.^[^ ^64^ ^]^ Previous studies have utilized immunohistochemistry staining to identify the presence of endothelial cells,^[^ ^26^, ^65^, ^66^ ^]^ and have quantified the ratio or volume occupied by cells within grafts,^[^ ^56^, ^58^, ^67^ ^]^ however these studies did not have the capacity to visualize the location of perfusable vessels in 3D within the graft or completely validate the functional status of these vessels.

Furthermore, to investigate endothelial cell infiltration and to detect smaller, capillary‐like vessels, whose lumen may be too narrow for perfusion with the radiopaque resin, CD31 expression was quantified on sectioned slides (Figure 6d). There was a significantly lower numbers of CD31‐positive cells present in photocross‐linked grafts compared with the native grafts at week 8, however this did not correlate with an increase in the number of vessel lumen or resin‐perfused vessels (Figure 6b,d). In the photocross‐linked grafts, more localized and reduced CD31 cell infiltration occurred; however, CD31 expression generally coincided with the formation of small vessel lumen, which were perfused by red blood cells (Figure 6d; Figure S6, Supporting Information). This has previously been demonstrated in bioprinted lipoaspirate as well as nanocellulose‐alginate hydrogels through staining of consecutive slides.^[^ ^65^ ^]^

Given that tissue oxygenation is an important driver of adipose tissue homeostasis, tissue remodeling and graft survival,^[^ ^68^ ^]^ the level of tissue ischemia within photocrosslinked grafts was evaluated using a pimonidazole hydrochloride hypoxia marker (which binds to cells with pO2 <10 mm Hg). There were no differences in hypoxia marker expression between native and photocross‐linked grafts, highlighting that the increased CD31 expression in native grafts did not correlate with increased tissue vascularization and oxygenation (Figure S7a,b, Supporting Information). There was a ≈2‐fold increase in graft hypoxia between two and eight weeks, suggesting graft vascularization was not complete and still ongoing (Figure S7, Supporting Information). Nevertheless, compared with previous studies at 8 weeks, a low hypoxic tissue area was observed, which may be due to the small diameter of adipocytes from the patient sample, and therefore better graft survival in all groups.^[^ ^57^ ^]^ Interestingly, the murine adipose tissue surrounding the implanted grafts expressed low levels of hypoxia, suggesting that native adipose tissue in this study had higher innate levels of hypoxia compared with other accounts regardless of engraftment status or degree of vascularization (Figure S8, Supporting Information).^[^ ^68^, ^69^, ^70^ ^]^

Overall, the extent of vascularization may have been underestimated in this study due to the immature functional capacity of macrophage populations in NOD *scid* mice.^[^ ^71^ ^]^ Early macrophage infiltration and polarization play a key support role in promoting angiogenesis in fat grafts by releasing angiogenic cytokines and degrading the ECM for sprouting of vascular endothelial buds.^[^ ^56^, ^72^, ^73^ ^]^ Despite this potential underestimation of graft vascularization, these data demonstrate that photocross‐linked and native adipose tissue grafts are equally supportive of the formation of larger vessels when implanted subcutaneously, and support the formation of both large vessels and smaller capillaries throughout the whole graft.

### 2.7 Shape Integrity and Controlled Delivery

Given that nutrient and oxygen diffusion is limited to distances of 100–200 µm,^[^ ^74^, ^75^ ^]^ vessels grow at ≈5 µm per hour during neovascularization,^[^ ^76^, ^77^, ^78^ ^]^ and that adipocytes are particularly sensitive to ischemic conditions,^[^ ^60^, ^79^ ^]^ lipoaspirate grafts must be applied and maintained in vivo as thin fibers or micro‐ribbons, with the smallest‐possible diameters to maximize cell survival and prevent tissue resorption. Therefore, increased structural control and shape retention is required to enhance the utility of lipoaspirate as a soft tissue filler.^[^ ^17^ ^]^

**Figure 7:**
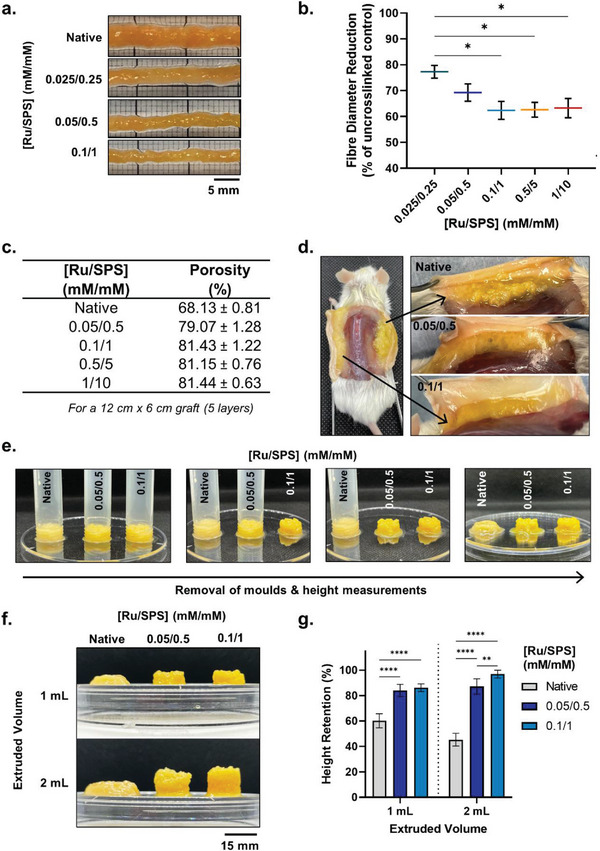
Shape integrity and stability of photocrosslinked lipoaspirate grafts. a) Representative images and b) quantification of extruded lipoaspirate fibers relative to the native controls. Error bars represent ± standard error. Asterisks denote significant differences as detected by one‐way ANOVA, * *p*<0.05. c) Mean theoretical porosity of clinically‐relevant sized grafts using native and photocrosslinked lipoaspirate of different intensities. Error bars represent ± standard error. d) Representative images of lipoaspirate fiber shape retention after subcutaneous injection. e) Stepwise overview of graft shape maintenance after extrusion into moulds. f) Representative images and g) quantification of extruded lipoaspirate grafts after 5 min. Values presented are the means of three individual patients. Error bars represent ± standard error. Asterisks denote significant differences as detected by two‐way ANOVA, ** *p*<0.01, ****p<0.0001.

To understand the potential of photocross‐linking for enhancing the structural properties of patient‐derived lipoaspirate for more controlled delivery, a range of fiber extrusion‐based experiments were conducted ex vivo and in vivo to mimic clinical fat grafting procedures. When patient‐derived lipoaspirate was photocross‐linked in 1 mL syringes and extruded as single fibers, there was a decrease in the diameter of the extruded fibers with increasing photoinitiator concentration, up until 0.1/1 mm mm ^−1^ (Ru/SPS) (**Figure** **7**a,b). Higher cross‐linking conditions (orange and red groups) did not result in further reductions of fiber diameter (Figure 7b). These results were consistent with the dityrosine analysis whereby there were no significant increases in dityrosine bond formation above 0.1/1 mm mm ^−1^ (Figure 1c), and therefore no further improvements in the shape integrity of lipoaspirate when extruded. Using these data, the porosity of a clinically‐sized graft (i.e., for an upper pole breast with 5 layers of a 10 cm width × 10 cm length graft) was calculated by theoretical means. At 0.05/0.5 mm mm ^−1^, the theoretical graft porosity increased ≈11% compared with native lipoaspirate grafts, and then a further ≈2% increase at 0.1/1 mm mm ^−1^, whereby graft porosity then remained constant with increased photoinitiator concentration (Figure 7c). This increased porosity in the photocrosslinked grafts translates to an increase in the surface area‐to‐volume ratio of the grafts, allowing for reduced revascularization time and greater potential for nutrient diffusion during vasculogenesis.^[^ ^24^, ^25^ ^]^ Furthermore, this retention of fiber integrity was demonstrated in vivo where photocross‐linked lipoaspirate was retained in thin fibers with high surface area subcutaneously, while native controls formed large boluses with low surface area (Figure 7d). These data demonstrate that visible light photocross‐linking of native lipoaspirate using optimized Ru/SPS photoinitiator concentrations results in greater control over extruded fiber diameter, which in turn leads to greater porosity of the graft through increased surface area‐to‐volume ratio and greater fiber control when injected into the body. Other studies have attempted to provide reduced fiber diameters and porosity through bioprinting lipoaspirate and nanocellulose composites, however an evaluation and quantification of shape integrity in vitro or in preclinical models was not investigated.^[^ ^26^, ^27^ ^]^

Finally, to understand structural maintenance over time, equal volumes of photocrosslinked lipoaspirate (and native non‐photocross‐linked controls) were extruded into plastic moulds and the height retention of the sample was measured five minutes after mould removal (Figure 7e,f). There was a significant improvement in height retention of photocross‐linked samples (at both 0.05/05 and 0.1/1 mm mm ^−1^ concentrations) compared with native controls (*p*<0.0001; Figure 7f,g). With increased extrusion volume from 1 to 2 mL, the height retention capability of native lipoaspirate further decreased from ≈60% to ≈45%, demonstrating that with increasing extrusion volume there is reduced shape maintenance of ex vivo grafts (Figure 7g). The reverse trend was observed with photocross‐linked grafts of 0.1/1 mm mm ^−1^, where height retention increased by ≈10% with doubling in extrusion volume (Figure 7g). Furthermore, there was a significant increase in height retention between the 0.05/0.5 mM mm−1and 0.1mM mm ^−1^ photocross‐linking intensities at the 2 mL extrusion volume; demonstrating greater shape maintenance with increased photoinitiator concentration (P = 0.0089; Figure 7g). These data highlight enhanced maintenance of shape integrity of photocross‐linked grafts compared with native lipoaspirate and, therefore, the increased capability for extruding larger grafts of controlled shape and size clinically without the reliance on scaffolding or support materials.

## 3 Conclusions

While photocross‐linking strategies have demonstrated the capability to improve and control the structural properties of polymer‐containing materials, an investigation on unmodified native living tissues has not been performed. In this study, we refined a Ru/SPS photocross‐linking system and applied it to complex patient‐derived lipoaspirate to assess whether it provides a viable strategy for enhancing the structural and mechanical properties of living tissues for clinical fat grafting applications. Dityrosine bonds were formed between ECM molecules present in the lipoaspirate, with the molar abundance of bonds measured by LC‐MS/MS and tailored by altering the concentration of photoinitiators added. At low phototoinitiator concentrations, clear structural changes could be observed within the ECM network, resulting in lipoaspirate explants that could be reproducibly cultured ex vivo, and hence could be used as ex vivo 3D adipose tissue models in future studies. The cells present within the lipoaspirate were viable and functional in ex vivo culture over two weeks, and when implanted subcutaneously in vivo the photocross‐linked grafts underwent neovascularization and demonstrated reduced variability in tissue resorption rates. The controlled response observed in vivo is likely due to an increase in graft shape integrity, where extruded fibers of photocross‐linked lipoaspirate had a reduced diameter, retained fiber integrity in vivo and formed more porous grafts when fibers were layered into larger grafts. This increase in surface area‐to‐volume ratio translates to an increase in the graft‐to‐recipient interface, which is an important finding given that clinically, poor graft survival and tissue resorption arises from limited nutrient diffusion into larger bolus grafts while the vasculature grows. Importantly, the strategy adopted in this study takes an autologous approach (using the patient's own fat), without the large regulatory hurdles and approval processes required for laboratory‐based implants. Based on outcomes of structural integrity, for this tissue type we propose an optimized ratio of 0.1/1 mm mm ^−1^ (Ru/SPS). Overall, this study provides a conclusive example of the opportunities of photocross‐linking technologies for delivering structural support to heterogenous patient‐derived materials and to ultimately achieve more desirable patient outcomes; warranting further research in other patient‐derived native tissue applications and highlighting the opportunity for designing advanced surgical tools to enhance delivery in a clinical setting.

## 4 Experimental Section

The systematic approach taken in this study involved 1) collection and photocross‐linking of fresh patient‐derived lipoaspirate, 2) quantification of dityrosine bond formation and ECM structure, 3) an investigation of the base rheological properties of photocross‐linked tissues, 4) measuring the cellular response to photocross‐linking in ex vivo culture, 5) an assessment of inflammatory cytokine production, 6) in vivo transplantation and tissue integration studies, 7) measuring graft vascularization, and 8) an evaluation of graft shape integrity under controlled delivery,

### Photocross‐linking of Patient‐Derived Material

*Lipoaspirate Sample Collection and Processing*: Lipoaspirate samples used in this study were collected from patients with informed consent undergoing fat grafting procedures within the Canterbury District Health Board. Ethical approval for the collection of patient material was obtained from the University of Otago Ethics Committee (reference number H20/068). To ensure consistent removal of infiltrate, contaminating blood products and oil across samples, all samples were washed with pre‐warmed Hank's balanced salt solution (HBSS), spun at 300 × g for 5 min and the cell pellet, upper oil and lower infiltrate‐layers removed. The resulting lipoaspirate was used within two hours for any cell experiments.

*Photocross‐linking Lipoaspirate*: A visible‐light cross‐linking strategy was used to enhance the mechanical and structural properties of patient‐derived lipoaspirate. Processed lipoaspirate samples were mixed with photoinitiators; tris(2,2‐bipyridyl)dichlororuthenium(II) hexahydrate (Ru) and sodium persulfate (SPS) at a 1:10 ratio.^[^ ^33^ ^]^ The Ru/SPS photocross‐linking system was chosen over other cross‐linking strategies, as it demonstrates an increased light penetration depth compared to other photocross‐linking systems (up to 3.6 cm), forming homogenous polymer networks at a depth of 1 cm.^[^ ^12^ ^]^ Cross‐linking intensities were varied with modification of photoinitiator concentrations from 0.05/0.5 to 1/10 mm mm ^−1^ (Ru/SPS). The range of photointitiator concentrations selected were guided by previous hydrogel applications,^[^ ^12^, ^31^ ^]^ and were refined based on desired structural changes in lipoaspirate observed. All samples were then photocross‐linked using visible light (400–450 nm) at 30 mW/cm^2^ for 3 min, in larger cubic (l = 7 mm, w = 7 mm, h = 3 mm) or smaller cylindrical (h = 1 mm, Ø = 5 mm) silicone moulds. Samples without the addition of photoinitiators were used as native controls.

### Dityrosine Detection, Quantification and ECM Visualization

A stable isotope dilution liquid chromatography tandem mass spectrometry (LC‐MS/MS) method was used for the detection and quantification of tyrosine and dityrosine in cross‐linked lipoaspirate samples. Isotopically‐labelled standards (tyrosine (^13^C_6_) and dityrosine (^13^C_18_, ^15^N_2_) were used to control experimental variations in recovery, matrix effect, and ionization. Standard calibration curves using the ratios of unlabeled to labelled analytes were used for quantification.

*Sample Preparation*: Fresh patient‐derived lipoaspirate was cross‐linked in square moulds as described above and snap frozen at −80 °C before further processing. To remove cellular contents (including lipids) and enrich for proteins, samples were freeze‐thawed in milliQ water and incubated in 0.5% SDS in Tris‐EDTA buffer with continuous agitation for 48 h at room temperature (RT). Samples were washed with 0.1% Triton X‐100 and then incubated in isopropanol for 48 h at RT. The decellularized samples were washed in milliQ water and snap frozen at −80 °C before further analysis.

*Acid Hydrolysis*: ECM samples (≈100 mg) were hydrolyzed in the presence of internal standards (15 nmol tyrosine (^13^C_6_) and 540 pmol dityrosine (^13^C_18_, ^15^N_2_)) in 6 m hydrochloric acid: trifluoric acid (2:1) containing 1% w/v phenol under a nitrogen‐rich environment at 150 °C for 30 min. To remove acid, samples were processed via solid phase extraction using Strata C18‐E (55 µm, 70 Å) cartridges. Samples were eluted with 80% (v/v) methanol, and eluents were dried using an Eppendorf centrifuge concentrator (Eppendorf, USA) and reconstituted in 0.1% (v/v) formic acid for analysis.

*LC‐MS/MS Method*: Standards and samples were analyzed using an online solid phase extraction method with a 6500 QTrap mass spectrometer coupled to an Infinity 1290 LC, utilizing a two‐position six‐port valve and a two‐position ten‐port valve. Standards and samples were stored on the autosampler tray at 5 °C before being injected on to a Strata‐X‐CW 25 µm on‐line extraction cartridge (20×2.0 mm) using 100% water containing 0.1% formic acid (Solvent A). The cartridge was then washed with 100% Solvent A for 1.5 min. Using 77% acetonitrile containing 0.1% formic acid (Solvent B) and 23% 100 mm ammonium formate (Solvent C), retained analytes were reverse eluted from the cartridge on to a pre‐equilibrated Imtakt Intrada Amino Acid column (150×3.0 mm). Isocratic elution was carried out for 9 min, followed by a linear gradient to 100% Solvent C over 30 sec. The cartridge and column were then flushed with 100% Solvent C for 1.5 min, before being re‐equilibrated with 77% Solvent B and 23% Solvent C for 3 min, followed by re‐equilibration of the cartridge alone with 100% Solvent A for 1 min. The column oven temperature was set to 40 °C and a flow rate of 0.25 mL min^−1^ was used throughout. Samples were analyzed and the areas of dityrosine and tyrosine peaks were determined using Analyst 1.7.2. All species were quantified by fragmenting the singly‐charged parent ion [M+H]+, monitoring the fragment ion resulting from the loss of CH_2_O_2_ in positive‐ion mode (Table S1, Supporting Information), measuring the area under the curve of the resulting peak, and then relating to standard calibration curves.

*Visualizing the ECM within Photocross‐linked Lipoaspirate*: Scanning electron microscopy (SEM) was used to visualise the ECM within freshly photocross‐linked lipoaspirate. Samples were fixed in 2.5% gluteraldehyde overnight at 4 °C, washed in 0.1 m sodium cacodylate buffer and postfixed in 1% osmium tetroxide for 1 h at room temperature. Fixed samples were washed in 0.1 m sodium cacodylate buffer, dehydrated in graded ethanol solutions (20%−100%) over 2 h and dried in a critical end‐point dryer using liquid carbon dioxide. Dried samples were mounted onto aluminium stubs using doubled‐sided carbon tape and sputter coated with 10 nm of gold/palladium (80:20 Au:Pd). Mounted samples were image using a Zeiss Sigma 300VP SEM operating in high vaccum mode.

### Characterization of Photocross‐linked Tissue Properties

*Tissue Mass‐Loss Analysis*: Mass‐loss studies were performed to assess the effect of photocross‐linking on lipoaspirate tissue retention. Six fresh patient‐derived lipoaspirate samples were photocross‐linked into square moulds as described above, and compared against uncross‐linked lipoaspirate controls. Three samples were treated with isopropanol for 24 h to remove the lipid fraction, weighed to obtain the initial wet mass (m_initial, t0_) after crosslinking and lyophilised immediately to obtain their dry weights (m_dry,t0_). The actual macromer fraction was calculated based on the Equation:

(1) $$\mathrm{Actual}\;\mathrm{macromer}\;\mathrm{fraction}=\frac{m_{\mathrm{dry},t0}}{m_{\mathrm{initial},t0}}$$

To allow for equilibrium swelling to be achieved and removal of unbound tissue, the remaining three samples were submerged in PBS and incubated overnight at 37 °C before treatment with isopropanol for 24 h. The samples were then lypholised and weighed to obtain dry weights (m_dry_). The tissue mass‐loss at day 1, i.e., the sol fraction, was calculated based on the following Equations:

(2) $$m_{\mathrm{initial},\mathrm{dry}}=m_{\mathrm{initial}\;}\times \mathrm{actual}\;\mathrm{macromer}\;\mathrm{fraction}$$

(3) $$\mathrm{Sol}\;\mathrm{fraction}=\frac{m_{\mathrm{initial},\mathrm{dry}}-m_{\mathrm{dry}}\;}{m_{\mathrm{initial},\mathrm{dry}}}\times 100\%$$

*Rheological Characterization*: The viscoelastic properties of cross‐linked lipoaspirate were measured using photorheology and rotational rheology to investigate the impact of photocross‐linking lipoaspirate on tissue mechanics. Measurements were obtained using a Physica MCR301 rheometer (Anton Paar, Germany), outfitted with an optical Peltier plate setup, plate–plate geometry (25 mm diameter) and a S1500 Spot UV Curing light guide and filter (400 – 500 nm). To ensure that oscillatory rheology measurements were made inside the linear viscoelastic regime of lipoaspirate, frequency (0.1–10 Hz, 10% strain) and amplitude (1%–20% strain, 1 Hz). Accordingly, all measurements were taken with a strain amplitude of 0.07%, 5 Hz frequency and 1 mm gap.

After reaching stable viscous properties, lipoaspirate samples were exposed to visible light (30 mW/cm^2^) for 30 sec – 3 min to induce photocross‐linking. To characterize viscoelastic and mechanical properties, the loss modulus, storage modulus and complex shear modulus were measured.

Viscosity and shear stress were recorded during a shear rate ramp between 0.1 and 100 s^−1^, at a constant temperature of 21 °C. Yield stress was determined from the shear rate ramps and was defined as the shear stress measured at onset of shear‐thinning.

### Lipoaspirate Ex Vivo Culture and Cell Response – Culture Conditions

The cell response toward photocross‐linking was assessed through establishing and maintaining patient‐derived lipoaspirate explants ex vivo over 2 weeks. Lipoaspirate was cross‐linked in cylindrical moulds as described above, and routinely cultured in 48 well‐plates in DMEM/F12 supplemented with 10% FBS, 1% penicillin and and 0.1% amphotericin B. Controls were comprised of lipoaspirate that was encapsulated within collagen hydrogels (final collagen concentration of 0.6 mg mL^−1^) without photopolymerization.

### DNA Quantification

For experiment normalization and to quantify the number of cells in lipoaspirate cultures, total DNA content was determined on day 0, 1, 7 and 14. Samples were digested at 56 °C in 1 mg mL^−1^ proteinase K dissolved in 10 mm Tris‐HCl and 1 mm EDTA (pH 7.5) for 16 h. Samples were treated with DNase‐free RNAase and total DNA was quantified using the CyQUANT® Cell Proliferation Assay Kit according to manufacturer's instructions. Data was normalized to sample wet weight before digestion.

### Metabolic Activity Quantification

As a measure of tissue health across ex vivo culture, the metabolic activity of lipoaspirate cultures was measured on day 1, 7 and 14. Briefly, each sample was incubated with media containing 10% alamarBlue® solution for 3.5 h at 37 °C. The fluoresence was measured at an excitation wavelength of 530 nm and emission wavelength of 590 nmn (according to manufacturer's instructions) and normalized by DNA content to account for potential variations in cell numbers.

### Glycerol Release Assay

To assess tissue function, lipid metabolism was measured using a Glycerol Release Assay (Sigma–Aldrich, USA). To quantify the secreted proteins, media samples were collected on day 1, 7 and 14 (after contact with the sample for 24 h) and were stored at −20 °C until the assays were performed. The assay was performed on triplicate samples (run in duplicates) as per manufacturers instructions and glycerol content was normalised by DNA content to account for potential variations in cell numbers.

### Inflammatory Cytokine Production – *Peripheral Blood Mononuclear Cell Isolation from Healthy Donors*

Healthy donors were recruited from the University of Otago. Collection of blood was approved by the Human Ethics Committee of the University of Otago and healthy donors consented under ethics number H18/088.

Whole blood was collected from donors in heparin coated tubes (Becton Dickinson, Franklin Lakes, NJ) and diluted at a 1:1 ratio with sterile PBS. Whole blood was layered over Ficoll (GE Healthcare) in SepMate‐50 tubes (StemCell Technologies, Vancouver, BC, Canada) according to the manufacturer's instructions. Cells were centrifuged at 1200 g for 10 mins at 4 °C. Supernatant with enriched peripheral blood mononuclear cells (PBMCs) were washed twice in PBS and centrifuged twice at 382 g for 4 mins. Cells were cryopreserved until use in the assay.

### LegendPlex Cytokine Analysis

Human PBMCs were stimulated in RPMI with either DynabeadsTM Human T‐activator CD3/28 beads (Thermo Fisher Scientific) at a 1:1 cell:bead ratio (positive control), ruthenium (0.05 and 0.1 mm), sodium persulfate (0.5 and 1 mm), or unstimulated in RPMI (negative control). Cells were incubated at 37 °C, 5% CO_2_ for 72 h. Cell supernatant was collected and incubated with fluorescent beads according to the Legendplex proinflammatory‐1 cytokine analysis kit (Biolegend, San Diego, CA, USA). Data were acquired using FacsDIVA software (Becton Dickinson) and analysis conducted using Flowjo (version 10.0.7, Tree Star) and Graphpad Prism 9 (GraphPad Softwarre Inc, San Diego, CA, USA).

### In Vivo Graft Transplantation – *Study Design and Overview*

An in vivo subcutaneous implantation model was used to asess the utility of lipoaspirate photocross‐linking on fat grafting outcomes. Ethical approval for the research protocol and procedures was obtained from the University of Otago Animal Ethics Committee (AUP‐21‐154), and was performed in accordance with the Animal Welfare Act and the ARRIVE guidelines for animal experimentation.^[^ ^80^ ^]^ Lipoaspirate, of human origin, was collected and transplanted into dorsal subcutaneous pockets in immuncompromised mice. Three experimental groups were investigated; 1) 0.05/0.5 mm mm ^−1^, 2) 0.1/1 mm mm ^−1^, and 3) non‐crosslinked native lipoaspirate controls. The implanted xenogeneic grafts was examined at two end‐points to reflect clinical follow‐up times; two (*n* = 4 per group) and eight weeks (*n* = 4 per group) post‐implantation. Vascularization was assessed by micro‐CT and tissue health was analyzed via immunohistological stainings (markers for adipocytes, vascular cells and hypoxia were quantified). Tissue structure, integration and remodeling was investigated via histological analysis and histomorphometry.

### Animal Husbandry and Surgical Procedures

Twelve male NOD *scid* gamma mice were housed together in IVC cages at the animal facility at the University of Otago and were enrolled in the study at 9 weeks of age. Throughout the study, animals received standard food pellets and water ad libitum, and were maintained under climate‐controlled conditions (≈22°C; 12 h light/12 h dark).

In each mouse, bilateral lipoaspirate grafts (of human origin) were implanted on the dorsal surface flanking the spine. Preoperatively, mice received Buprenorphine (Temgesic, 0.1 mg kg^−1^ bodyweight) via subcutaneous injection; and Lidocaine (4 mg kg^−1^ bodyweight) via subcutaneous injection at the site of incision. Two dorsal subcutaneous pockets (15 mm deep) were created from 6 mm dorsal incisions and blunt dissection while under general aneasthesia (2%–3% isoflurane in oxygen), as previously described. Within each subcutaneous pocket a 0.3‐cc graft from one of the experimental groups (native control, 0.05/0.5 or 0.1/1 mm mm ^−1^) was implanted (*n* = 4 per group) – resulting in each mouse containing two different experimental groups. The skin incision was closed with three stiches using Mono Q® resorbable 5‐0 sutures. For pain relief, Carpofen (5 mg kg^−1^ bodyweight) was administered subcutaneously for three days post‐surgery.

Mice were euthanized at two and eight weeks after surgery via an intraperitoneal overdose of Pentobarbital (Pentobarb,150 mg kg^−1^ bodyweight). To visualize hypoxic regions downstream, 1 h before euthanasia, pimonidazole HCl (Hypoxyprobe™^−1^, 60 mg kg^−1^ bodyweight) was injected intravenously through the tail.

### Lipoaspirate Implant Preparation

Lipoaspirate was processed and photocross‐linked to enhance the mechanical and structural properties as described for in vitro experiments with a few variations. For preparation of each graft, 300 µL of the lipoaspirate samples (± photoinitiators) were loaded into 1 mL syringes and photocross‐linked using visible light (400 – 450 nm) at 30 mW cm^2^ for 3 min. This was achievable given the light penetration depth of this photocross‐linking system.^[^ ^12^ ^]^ Nevertheless, to ensure homogeneous cross‐linking mid‐way through light‐irradiation, the syringes were rotated 180 degrees and the subsequent side was photocrosslinked. The prepared grafts were implanted within 5 min of photocross‐linking.

### Histology and Immunohistochemistry

Fixed samples (both ex vivo and in vivo) were processed in a vacuum tissue processer, embedded in paraffin and sliced into 8 µm thick sections. Before staining, samples were baked at 60 °C overnight, deparaffinized with xylene and rehydrated using graded ethanol (100% – 70% EtOH). To histologically asses the whole graft, tissue sections were analyzed at different depths of the graft (three sections, 200 µm apart) and averaged to obtain the percentage of stain area.

Overall tisue structure and quality was evaluated using haematoxylin and eosin staining (Richard‐Allen Scientific). For perilipin (*ab3526*), CD31 (*ab281583*) and hypoxia (Hypoxyprobe©) staining, endogeneous peroxidase activity was blocked by incubating samples in 0.3% H_2_O_2_ for 10 min, followed by aspecific protein blocking with 5% BSA‐PBS for 30 min at room temperature. Antigen retrieval, and primary, secondary and isotype antibody dilutions are outlined in Table S3 (Supporting Information). Rabbit isotypes were used as negative controls at concentrations matched with those of the primary antibodies (Table S3; Figure S1, Supporting Information). Slides were visualized by DAB oxidation and counterstained with hematoxylin to visualize cell nuclei. Stained slides were washed, dehydrated in graded ethanol (70%–100% EtOH) and mounted in DPX. Slides were viewed and imaged at 20× using an Aperio CS2 Slide scanner. Three sections per sample were stained and positive cells were analysed using Adobe photoshop C6 and ImageJ software (version 6.1 Fiji, National Institutes of Health). The operator was blinded during the staining, acquisition and counting phases.

### Assessing Vascularization in Photocross‐linked Grafts – *Vessel Perfusion in Graft Explants*

Vascularization was assessed at the two‐ and eight‐week end‐points with the use of micro‐CT angiography. Immediately after overdose with barbiturates (Pentobarb) and assessment of pedal withdrawal reflex, a lateral incision was made through the integument and abdominal wall, and the diaphragm was cut along the entire length of the rib cage to expose the pleural cavity. The rib cage was cut, and sternum lifted away to expose the heart, and a cardiac puncture was performed. Specifically, a 23G catheter was inserted into the left ventricle and an incision was made in the right atrium to create an outlet for blood (exsanguination) and the solutions perfused in the circulatory system. Heparinized PBS (20 U mL^−1^), followed by 10% neutral buffered formalin, was perfused using a peristaltic pump at 5 mL min^−1^ for 2 min. After fixation, 5 mL of radiopaque contrast agent (Microfill MV‐122, Flow Tech) was prepared and perfused as per manufactures instructions, and the compound was polymerized for 6 h at 4 °C. Following Microfill polymerization, grafts were explanted and post‐fixed in 10% neutral buffered formalin for 24 h.

### Visualizing Explanted Grafts using Micro‐CT

To measure the volume of perfused vessels within each graft, implants were scanned individually using micro‐CT. Instrument settings were kept constant for each sample (Table S2, Supporting Information). Volumes of interest were segmented with a global threshold, and vessel volume was measured using the image processing software 3D slicer (3D slicer). 3D reconstructions of the grafts with blood vessels were based on the micro‐CT data and created using Meshlab.

### Shape Integrity in Photocross‐linked Grafts – *Fiber Diameter Quantification*

A fiber extrusion experiment was conducted to evaluate the effects of photocross‐linking on the structural properties of patient‐derived lipoaspirate. Lipoaspirate was cross‐linked in syringes as described above at photoinitiator concentrations from 0.025/0.25 – 1/10 mm mm ^−1^ (Ru/SPS). Upon completion of light exposure, 1 mL of photocross‐linked lipoaspirate was hand‐extruded into straight fibers onto petri dishes. The extruded fibers were immediately viewed under an Olympus SD30 dissection microscope fitted with a Canon Powershot A630 camera and 10 fields‐of‐view (FOV) were imaged per condition. Native lipoaspirate without photoinitiators were also extruded as non‐photocross‐linked controls. For each FOV image, the fiber diameter was measured at three positions along the fiber (total of 30 measurements per condition). The percent reduction in fiber diameter was determined using the formula:

(4) $$\begin{array}{c} \left(1-\frac{\left(Native\;lipoaspirate\;Ø-photocrosslinked\;lipoaspirate\;Ø\right)}{Native\;lipoaspirate\;Ø}\right)\times 100 \end{array}$$

### Height and Shape Retention Experiments

To further assess the capability of photocross‐linking for improving the structural capacities of lipoaspirate, photocrosslinked lipoaspirate (0.05/0.5 and 0.1/1 mm mm ^−1^) and native controls were extruded into tall moulds and the the height retention measured 5 min after mould removal. The percent heigh retention was determined using the formula:

(5) $$\begin{array}{c} \left(\frac{\left(Initial\;sample\;height\;in\;mould-Sample\;height\;after\;5\;min\right)}{Initial\;sample\;height\;in\;mould}\right)\times 100 \end{array}$$

### Graft Porosity Evaluation

As a measure of surface area‐to‐volume ratio in clinically‐sized grafts, the porosity of a 10 cm by 10 cm graft with five layers (like the size of an upper pole breast implant) was calculated. The porosity of cross‐linked and control layered grafts was evaluated following Landers et al. theoretical approach^[^ ^81^ ^]^ :

(6) $$P\;=\;1-\;\frac{V_{graft}}{V_{cube}}=\;1-\frac{\pi }{4}\;\times \;\frac{1}{\left(\frac{d_{2}}{d_{1}}\right)}\;\times \frac{1}{\left(\frac{d_{3}}{d_{1}}\right)}\times 100$$

Where, *P* is the graft porosity, *d_1_* is the fibre diameter, *d_2_* is the fibre spacing and *d_3_* is the layer thicknesss of the graft.

### Statistical Analysis

All experiments were performed in triplicate for each cross‐linking condition and repeated across three individual patients. The results are expressed as mean ± standard error of the mean when required. Using GraphPad Prism 6 (GraphPad Prism 6, San Diego, CA, USA), groups of data were compared by One‐way ANOVA or Kruskal‐Wallis test (when normality assumption was not met – PBMC LegendPlex assay), with a P value of <0.05 considered statistically significant. Where relevant, post hoc Tukey's HSD tests were used for pairwise comparisons following ANOVA.

## Conflict of Interest

The authors declare no conflict of interest.

## Supporting information

Supporting Information

## Data Availability

The data that support the findings of this study are available on request from the corresponding author. The data is not publicly available due to privacy or ethical restrictions.
