# Supplementary material for: Clinical Applicability of Visible Light‐Mediated Cross‐linking for Structural Soft Tissue Reconstruction
Source: Adv Sci (Weinh). 2023 Jul 9;10(26):2300538. doi: 10.1002/advs.202300538 (PMC10502829; doi:10.1002/advs.202300538)
Supplement: Supplementary file 1 — Supporting Information [file ADVS-10-2300538-s001.pdf]

ADVANCED SCIENCE
Open Access

Supporting Information

for *Adv. Sci.*, DOI 10.1002/advs.202300538

Clinical Applicability of Visible Light-Mediated Cross-linking for Structural Soft Tissue Reconstruction

*Gretel Major, Alessia Longoni, Jeremy Simcock, Nicholas J Magon, Jessica Harte, Boushra Bathish, Roslyn Kemp, Tim Woodfield and Khoon S Lim**

Supplementary Information:

Table S1. The m/z values for the singly-charged parent and fragment ions, and the optimised parameters that were used to quantify each analyte in LC-MS/MS experiments. DP (declustering potential), EP (entrance potential), CE (collision energy), CXP (cell exit potential).

Analyte	Parent (m/z)	Fragment (m/z)	DP	EP	CE	CXP
Tyrosine (H+)	182.08	136.08	99	10	25	13
Tyrosine ($^{13}C_6$) (H+)	188.10	142.10	99	10	25	13
Dityrosine (H+)	361.14	315.13	76	6	23	37
Dityrosine ($^{13}C_{18}$,$^{15}N_2$) (H+)	381.19	334.19	76	6	23	37
Trityrosine (H+)	540.20	494.19	88	6	32	20

Table S2. Micro-CT image-acquisition parameter settings

Parameter	Setting
Camera	Hamamatsu 10Mp
Camera Pixel size	11.45 um
Source Voltage	29 kV
Source Current	169 uA
Image pixel size	12.7 um
Rotation Step	0.5 degrees
Averaging	2 frames
Scan time	37 min

Table S3. Immunohistochemistry antibodies and antigen retrieval.

Primary Antibody	Final Concentration	Antigen Retreival	Secondary Antibody	IgG Isotype Dilution
Anti-Perilipin *ab3526* (1:200)	5 µg/mL incubation O/N at 4°C	Boiling at 95°C in Tris-EDTA buffer (pH 9) for 20 min	Goat Anti-Rabbit IgG H&L (HRP) *ab205718* (1:5000) 400 ng/mL Incubation 1 h at RT	1:335
Anti-CD31 *ab281583* (1:2000)	240 ng/mL incubation 30 min at RT			1:7220
Anti-Piramidazole-FITC (1:100)	5 µg/mL incubation 1 h at RT	Boiling at 95°C in 10 mM citrate buffer (pH 6) for 20 min	(1:100) incubation 30 min at RT	No primary Antibody

Table S4. Lipoaspirate graft resorption after eight weeks *in vivo*, as measured using micro-CT.

Condition (mM/mM Ru/SPS)	Graft Volume (mm^3 ± SD)	Resorption (%)
Native control	236.8 ± 35.1	21.1
0.05/0.5	244.2 ± 30.4	18.6
0.1/1	221.3 ± 18.3	26.2

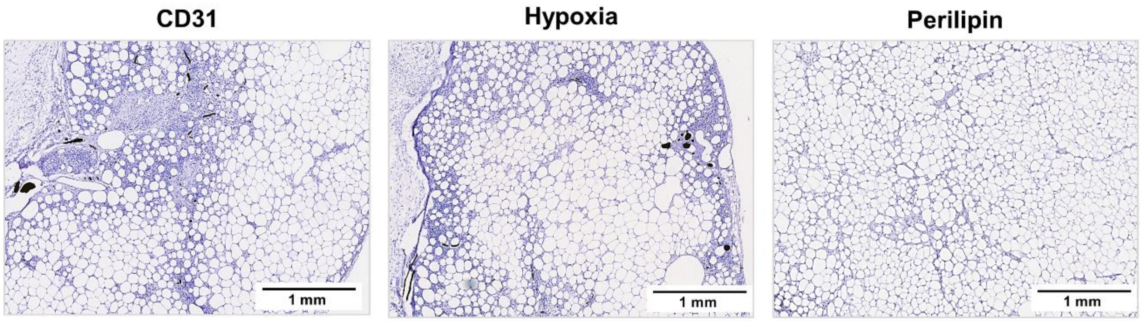

Figure S1. Isotype controls for IHC stains: CD31, pimonidazole (hypoxia) and perilipin. Representative images taken at 10× magnification.

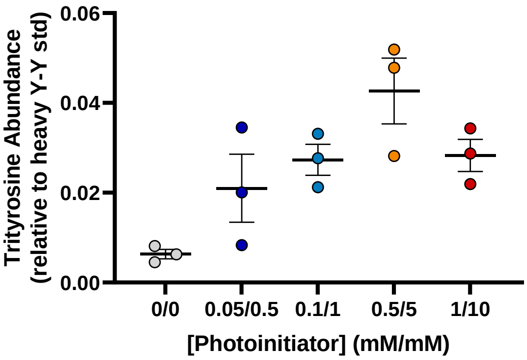

Figure S2. Quantification of trityrosine abundance in isolated lipoaspirate ECM samples calculated relative to isotopically labelled dityrosine. Values presented are the means of three individual patients (with each patient sample performed in triplicate). Error bars represent ± standard error.

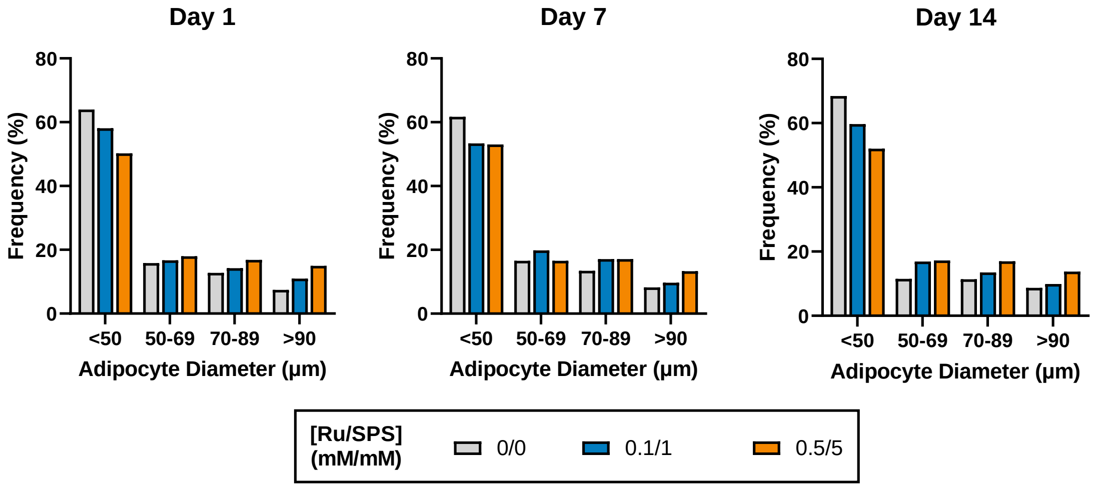

Figure S3. Adipocyte diameter size distribution of control (grey), low- (blue) and high- (orange) photocrosslinked samples cultured *ex vivo* over 14 days.

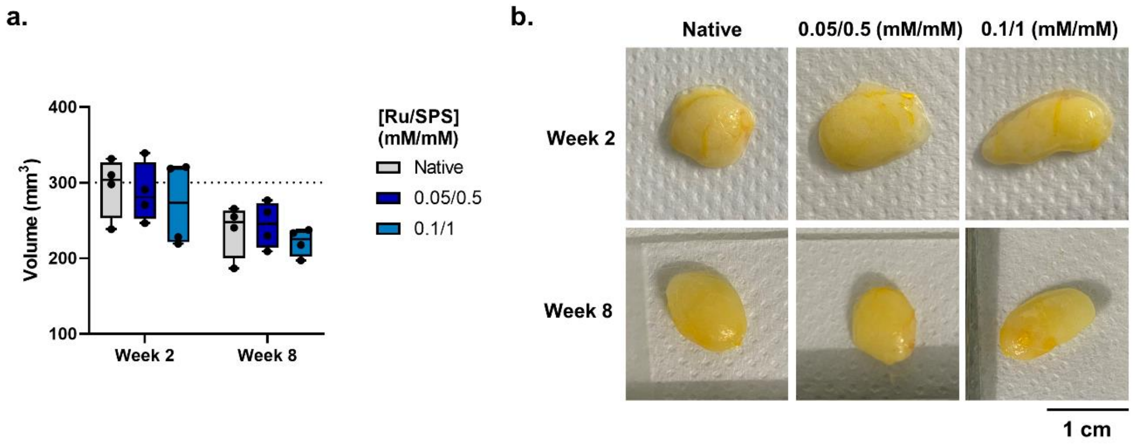

Figure S4 Graft volume of native and photocrosslinked grafts implanted *in vivo* after two and eight weeks. **a)** Volume quantification using micro-CT and **b)** macro-images of explanted grafts.

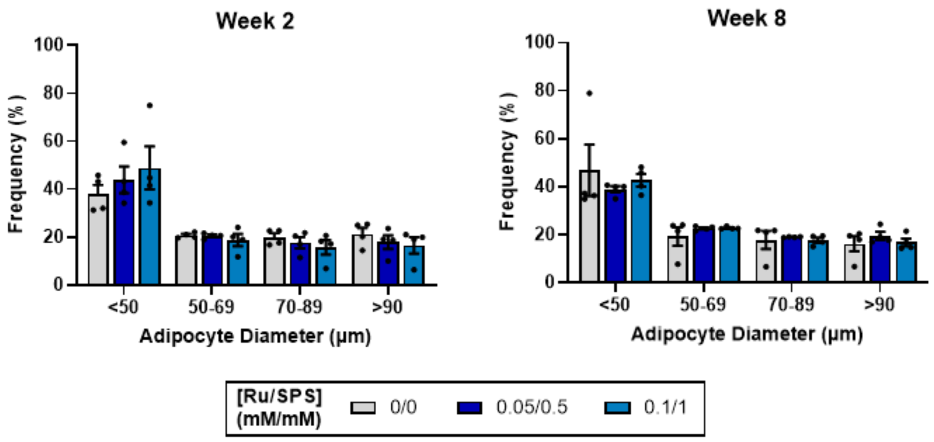

Figure S5. Adipocyte diameter size distribution of native and photocrosslinked grafts implanted *in vivo* after two and eight weeks.

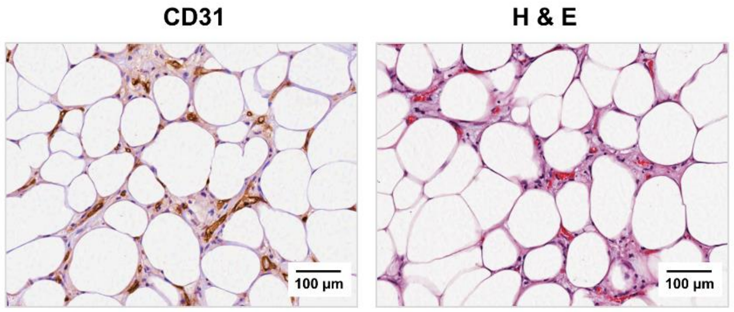

Figure S6. Representative images of red blood cell perfused capillaries in photocrosslinked grafts (0.1mM/1mM Ru/SPS). Consecutive images of CD31 IHC staining and haematoxylin and eosin staining at 10× magnification.

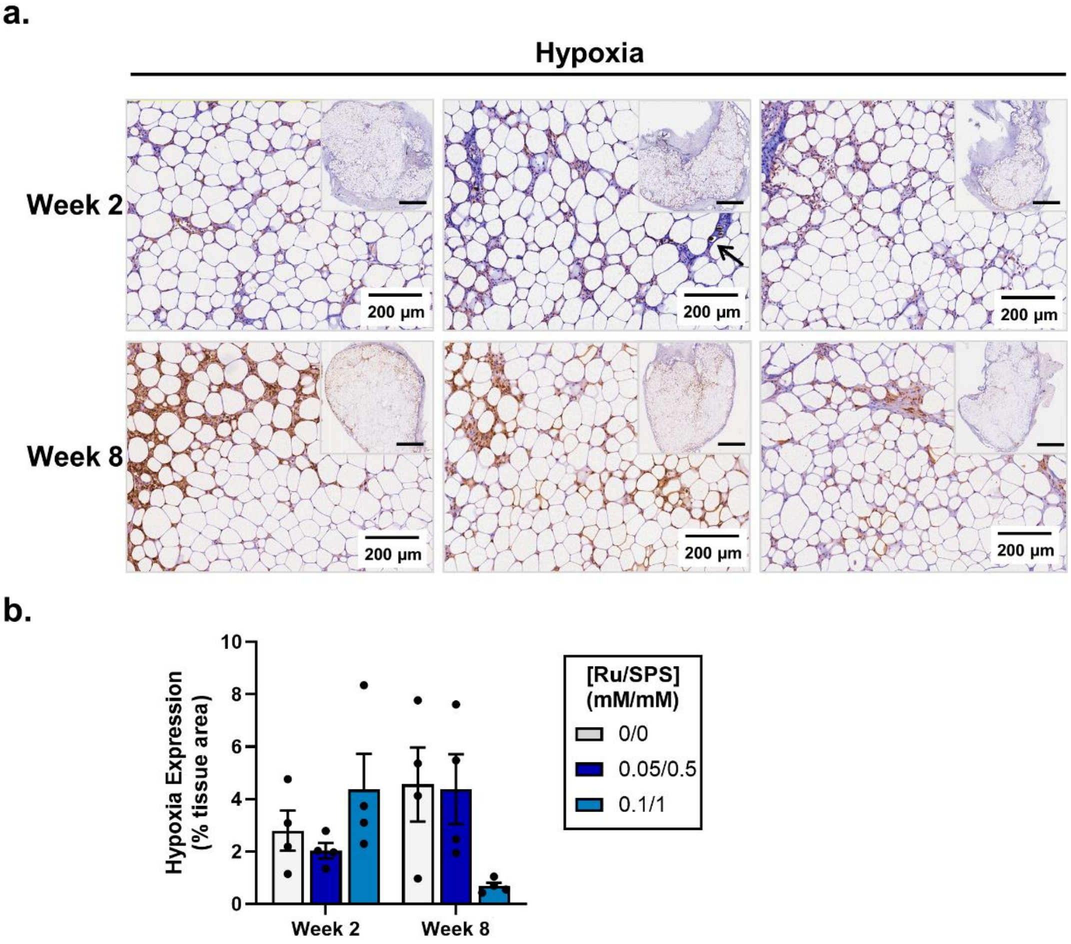

s

Figure S7. Graft hypoxia marker expression. **a)** Representative images of pimonidazole (hypoxia) IHC sections at 10× magnification. Inset scale bars are 2 mm **b)** Hypoxia expression quantification of photocrosslinked grafts after two and eight weeks. Values presented are the means of four mice implanted with the same patient sample (with quantification performed on triplicate slides per graft). Error bars represent ± standard error.

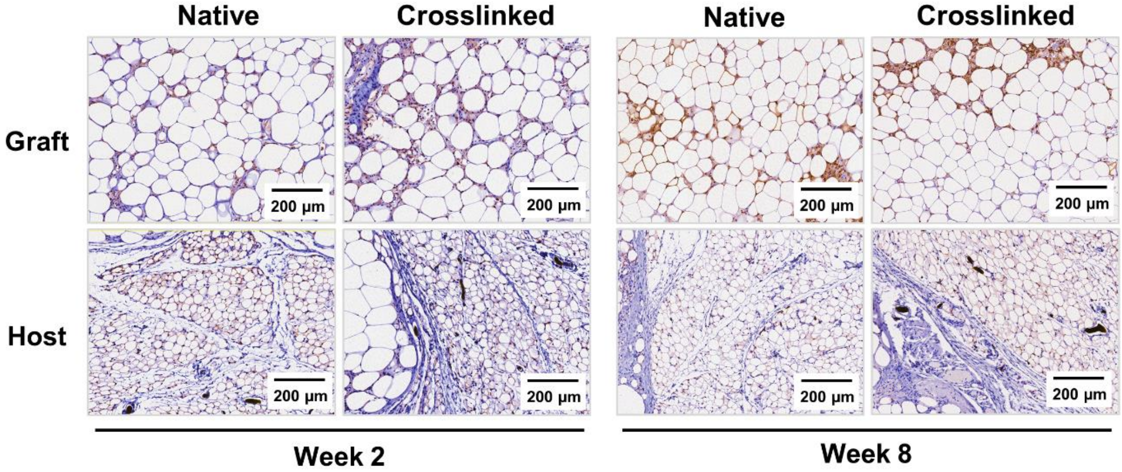

Figure S8. Representative pimonidazole (hypoxia) IHC sections in native and photocrosslinked grafts, and the surrounding murine adipose tissue.

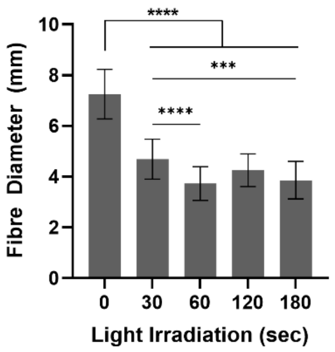

Figure S9. Lipoaspirate fibre diameter with increasing light irradiation dose (sec) when photocrosslinked with 0.1/1 mM/mM (Ru/SPS). Error bars represent ± standard error. Asterisks denote significant differences as detected by one-way ANOVA, * $p<0.05$
